# Treatment of Inflammatory Bowel Disease with Drugs Targeting PANoptosis: A Comprehensive Review

**DOI:** 10.3390/biomedicines14010148

**Published:** 2026-01-11

**Authors:** John K. Triantafillidis, Stavros Karakatsanis

**Affiliations:** Metropolitan General Hospital, Holargos, 155 62 Athens, Greece; karakatsanisendoscopy@outlook.com.gr

**Keywords:** PANoptosis, apoptosis, pyroptosis, inflammatory bowel disease, ulcerative colitis, Crohn’s disease, biologic agents

## Abstract

**Background:** Inflammatory Bowel Disease (IBD) involves a complex interplay between immune dysregulation and intestinal barrier failure. Traditional views focused on individual cell death pathways, but the emerging concept of PANoptosis—a coordinated inflammatory cell death involving apoptosis, necroptosis, and pyroptosis—offers a more holistic understanding of IBD pathogenesis. **Objective:** This review evaluates the role of PANoptosis in IBD, identifies key molecular triggers (such as the ZBP1-ADAR1 axis), and discusses the therapeutic potential of targeting this process. **Methods:** We analyzed recent literature and clinical trial data regarding programmed cell death (PCD) inhibitors and natural compounds in IBD models. **Results:** Preclinical data suggest that targeting PANoptotic regulators like RIPK1 and ZBP1 can restore barrier integrity. However, clinical translation remains challenging; for instance, while targeting pyroptosis via IL-1/IL-18 (Anakinra) showed promise in theory, clinical results in IBD have been disappointing. Furthermore, RIPK1 inhibitors such as GSK2982772 have failed to meet primary endpoints in Phase 2 trials. **Conclusions:** PANoptosis is a “hot” therapeutic target, but successful treatment likely requires combination therapies or “PANoptosome” specific modulators rather than single-pathway inhibition.

## 1. Introduction

Inflammatory Bowel Disease (IBD) is a heterogeneous group of disorders that includes ulcerative colitis (UC) and Crohn’s disease (CD). It is characterized by damage to the intestinal mucosa, chronic inflammatory infiltrate, and increased production of proinflammatory cytokines. Despite therapeutic advances over the last decades with the use of so-called biological agents, a significant proportion of patients remain resistant to treatment [1].

The epithelial cells of the intestinal mucosa, in addition to their contribution to the function of absorption and digestion of nutrients, also function as a protective barrier against the (harmful) content of the intestinal lumen and the intestinal flora. In contrast, through the mucosal immune cells located in the lamina propria, it maintains intestinal homeostasis. These intestinal cells form a layer of specialized epithelial cells (IECs) interconnected by tight junctions (the intestinal epithelial barrier). The integrity of this epithelial barrier is of primary importance for the homeostasis of the organism. The pronounced death of IECs increases intestinal permeability, leading to bacterial translocation into the mucosal lamina propria and triggering an inflammatory response that is perpetuated. Changes in the programmed cell death (PCD) model of the intestinal epithelium may trigger an inflammatory process [2]. The pathogenesis of IBD is primarily characterized by extensive damage or death of intestinal epithelial cells, along with abnormal activation or dysregulation of immune cell death and the release of various inflammatory cytokines.

Intestinal homeostasis depends on two factors related to enterocytes: survival and functionality. The processes of PCD, which include apoptosis, pyroptosis, autophagy, ferroptosis, necroptosis, and neutrophil extracellular traps, are essential to the pathogenesis of IBD, as they contribute to the death of intestinal epithelial and immune cells. While apoptosis is considered an immunologically silent cell death, pyroptosis and necroptosis are highly inflammatory forms. Early research focused heavily on apoptosis, however it is now recognized that classical apoptotic bodies are rarely the dominant feature in human UC or CD biopsies, except in specific cases, like autoimmune enteropathy. Instead, the tissue damage seen in IBD patients more closely resembles lytic forms of PCD. PANoptosis emerges as a more accurate framework, as it captures the “cross-talk” between pathways. It is organized by the PANoptosome, a molecular scaffold that allows the simultaneous activation of caspases (pyroptosis/apoptosis) and RIPK kinases (necroptosis) [3].

In this comprehensive review, we describe data that highlight PANoptosis as a key process in IBD and a promising therapeutic target in the near future.

## 2. Programmed Cell Death (PCD)

PCD is a highly regulated cellular process in which specific genes are activated in response to internal or external environmental stimuli, orchestrating the orderly death of the cell. It corresponds to a precise and physiological self-destructive process within the cell that is essential for maintaining homeostasis and regulating disease development. An imbalance in PCD can trigger inflammation and an immune response. So far, several PCD modes have been described, including apoptosis, necroptosis, pyroptosis, ferroptosis, entotic cell death, NETosis, parthanatos, lysosome-dependent cell death, autophagy-dependent cell death, alkaliptosis, oxeiptosis, cuproptosis, disulfidocytosis, and PANoptosis [4,5,6]. Overall, the number of recognized models is constantly expanding as scientific research uncovers new molecular mechanisms.

The predominant forms of PCD include apoptosis, necrosis, and pyroptosis. It is argued that the various PCD pathways exhibit significant cross-regulatory interactions that collectively influence cellular outcomes. The major PANoptosis pathways are shown in Table 1 and discussed below.

### 2.1. Apoptosis

Apoptosis represents a tightly regulated and predominantly non-inflammatory form of PCD that plays a central role in intestinal epithelial homeostasis. In the intestinal mucosa, apoptosis occurs primarily in IECs, particularly at the villus tip or surface epithelium, where it contributes to physiological epithelial turnover and barrier renewal. Under inflammatory conditions, however, excessive or dysregulated apoptosis may compromise epithelial integrity and increase mucosal permeability.

Apoptosis can be initiated through either the extrinsic or intrinsic (mitochondrial) pathway. The extrinsic pathway is triggered by the binding of death ligands, such as tumor necrosis factor (TNF) or Fas ligand, to their corresponding death receptors on the plasma membrane. This interaction leads to recruitment of adaptor proteins, including FADD or TRADD, and formation of the death-inducing signaling complex (DISC), which activates caspase-8. Activated caspase-8 subsequently induces the effector caspases-3 and -7, resulting in controlled cellular dismantling without release of intracellular danger signals.

The intrinsic apoptotic pathway is activated by intracellular stressors relevant to IBD, including oxidative stress, hypoxia, endoplasmic reticulum stress, and exposure to proinflammatory cytokines. These stimuli induce activation of pro-apoptotic members of the B-cell lymphoma-2 (Bcl-2) protein family, leading to mitochondrial outer membrane permeabilization and release of cytochrome c. Cytochrome c associates with apoptotic protease-activating factor-1 (Apaf-1) to form the apoptosome, triggering caspase-9 activation and downstream activation of caspases-3/7 [7,8].

Importantly, in the inflamed intestinal mucosa, apoptosis is not an isolated process but represents an early and potentially reversible response to epithelial stress. When apoptotic signaling is impaired or overwhelmed—particularly through inhibition of caspase-8—cells may switch toward alternative, highly inflammatory forms of PCD, such as necroptosis or pyroptosis.

### 2.2. Necroptosis

The word necroptosis is a compound of the ancient Greek words “νεκρός” (“nekros” = dead) and “πτώσις” (“ptosis” = fall), meaning “fall (in) death”. Necroptosis is a lytic and proinflammatory form of programmed cell death that typically emerges when apoptotic pathways are blocked or insufficient. In the intestinal mucosa, necroptosis has been most prominently described in IECs under conditions of chronic inflammation, where persistent TNF signaling and caspase-8 inhibition favor necroptotic execution. Understanding the pathogenesis of necroptosis in IBD will enable the development of additional therapeutic approaches for the illness [9].

Morphologically, necroptotic cells exhibit mitochondrial swelling, plasma membrane rupture, and release of intracellular contents, including damage-associated molecular patterns (DAMPs), which amplify mucosal inflammation. Caspase-8 functions as a critical molecular checkpoint determining cell fate: when active, it promotes apoptosis and suppresses necroptosis; when inhibited or absent, necroptotic signaling predominates.

Necroptosis is initiated following engagement of death receptors or pattern recognition receptors, leading to activation of receptor-interacting protein kinase-1 (RIPK1). RIPK1 subsequently interacts with RIPK3 to form the necrosome complex. Within this complex, RIPK3 phosphorylates mixed lineage kinase domain-like protein (MLKL), which oligomerizes and translocates to the plasma membrane, where it forms pores and induces membrane rupture. This process results in the extracellular release of DAMPs and proinflammatory mediators, fueling immune cell recruitment within the lamina propria [10,11,12].

Evidence from human studies supports a role for necroptosis in IBD pathogenesis. Increased expression and phosphorylation of RIPK3 and MLKL have been detected in colonic biopsies from patients with UC, suggesting active necroptotic signaling particularly within the epithelial compartment. Spatially, epithelial necroptosis acts as a potent trigger for secondary immune activation in the lamina propria, thereby linking epithelial injury to sustained mucosal inflammation.

### 2.3. Pyroptosis

“Pyroptosis” is a term composed of the union of two separate ancient Greek words, namely “πυρ” (-pyr = fire) and “πτώσις” (-ptosis = fall), which together mean “fire fall”. Pyroptosis is a highly inflammatory form of programmed cell death characterized by gasdermin-mediated membrane pore formation and rapid cell lysis. In contrast to apoptosis and necroptosis, pyroptosis occurs predominantly in innate immune cells of the lamina propria, such as macrophages and dendritic cells, although increasing evidence suggests that IECs may also undergo pyroptosis under severe inflammatory stress [13].

Pyroptotic cell death is executed through activation of inflammatory caspases, including caspase-1 (canonical pathway) and caspases-4/5/11 (non-canonical pathway), resulting in cleavage of gasdermin D (GSDMD). Morphologically, pyroptotic cells display cellular swelling, membrane rupture, and release of intracellular contents, including mature proinflammatory cytokines, which strongly amplify local immune responses.

Within the intestinal mucosa, pyroptosis integrates temporally downstream of epithelial barrier disruption and microbial translocation. Loss of epithelial integrity—whether driven by apoptosis or necroptosis—facilitates exposure of lamina propria immune cells to pathogen-associated molecular patterns (PAMPs) and DAMPs, thereby triggering inflammasome activation and pyroptotic cell death [14]. Moreover, dysregulated colorectal microflora also contributes to activating the pyroptotic pathway in UC patients, initiating an inflammasome- and gasdermin-dependent inflammatory cell death process [15].

#### 2.3.1. Activation of the NLRP3 Inflammasome

Among the various inflammasomes, the NLRP3 inflammasome is the most extensively studied in IBD. Its activation is a tightly regulated two-step process:

Priming: Initial exposure to microbial components (e.g., lipopolysaccharide) or inflammatory cytokines (e.g., TNF-α) activates nuclear factor-κB (NF-κB), leading to transcriptional upregulation of NLRP3 and pro-caspase-1.

Activation: Subsequent exposure to danger signals relevant to IBD pathophysiology—including extracellular ATP, mitochondrial reactive oxygen species, potassium efflux, or epithelial barrier disruption—induces NLRP3 oligomerization, recruitment of the adaptor protein ASC, and activation of caspase-1 [16].

#### 2.3.2. Caspase-1 and GSDMD

Activated caspase-1 serves as the central executioner of pyroptosis and mediates two key downstream effects. First, it cleaves the inactive precursors pro-IL-1β and pro-IL-18 into their biologically active forms, IL-1β and IL-18, both of which are markedly elevated in the intestinal mucosa of patients with IBD and contribute to disease severity.

Second, caspase-1 cleaves GSDMD, releasing its N-terminal fragment, which inserts into the plasma membrane and forms large pores approximately 10–20 nm in diameter. These pores permit ionic flux, water influx, and eventual osmotic lysis of the cell, leading to the extracellular release of cytokines and intracellular contents. This process establishes pyroptosis as a critical amplifier of mucosal inflammation, linking innate immune activation in the lamina propria with epithelial injury and perpetuation of intestinal inflammation [17].

**Figure 1:**
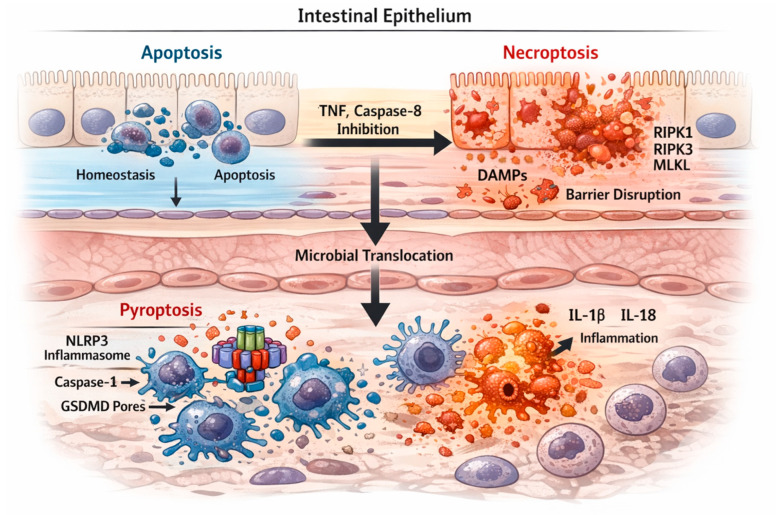
Temporal and spatial integration of apoptosis, necroptosis, and pyroptosis in the intestinal mucosa during IBD. A schematic cross-section of the intestinal mucosa depicting IECs in the upper compartment and lamina propria immune cells below. Under physiological or early inflammatory stress, IECs primarily undergo apoptosis, a non-lytic and non-inflammatory process that supports epithelial turnover and barrier maintenance. In the context of persistent inflammatory signaling, particularly sustained TNF exposure and inhibition of caspase-8, apoptotic signaling is diverted toward necroptosis in IECs. Necroptosis is mediated by RIPK1–RIPK3 complex formation and MLKL activation, resulting in epithelial membrane rupture, release of damage-associated molecular patterns (DAMPs), and barrier disruption. Loss of epithelial integrity facilitates microbial translocation and accumulation of pathogen-associated molecular patterns (PAMPs) and DAMPs within the lamina propria. These signals activate innate immune cells, including macrophages and dendritic cells, leading to assembly of the NLRP3 inflammasome, caspase-1 activation, and gasdermin D–mediated pyroptosis. Pyroptotic immune cell death is accompanied by release of IL-1β and IL-18, which further amplify mucosal inflammation and perpetuate epithelial injury. Together, these pathways form a dynamic, interconnected network linking epithelial stress responses with innate immune activation and chronic intestinal inflammation.

Figure 1 provides a spatial and temporal framework for understanding the coordinated activation of programmed cell death pathways in IBD. As illustrated in this Figure, epithelial apoptosis precedes inflammatory cell death pathways, with caspase-8 acting as a key molecular checkpoint.

### 2.4. PANoptosis

PANoptosis—a relatively recent term in the literature derived from the ancient Greek words “παν” (“pan” = total) and “πτώσις” (“ptosis” = fall)—describes a comprehensive form of PCD, which incorporates the characteristics and molecular pathways of three distinct but overlapping mechanisms: apoptosis, pyroptosis, and necroptosis [13]. The coupling of these pathways leads to a form of cell death characterized by high inflammatory activity and active participation in the pathophysiology of IBD. This mechanism is fundamental in the intestinal mucosa, where uncontrolled PANoptosis leads to increased barrier disruption, systemic release of inflammatory cytokines (IL-1β, IL-18), and chronic self-perpetuating inflammation. It is mediated by multiprotein complexes called PANoptosomes (ZBP1-, AIM2-, RIPK1-, and NLRP12-PANoptosomes), which are generated in response to specific stimuli associated with various pathogens or damage. This process contributes to tissue homeostasis by removing damaged cells, while enhancing the immune/defense system through the release of inflammatory cytokines and damage-related molecular patterns. In particular, in UC and CD, PANoptosis leads to epithelial cell death, disrupts the mucosal barrier, and promotes the inflammatory process [18]. During the course of IBD, apoptosis, necrosis, and pyroptosis occur; therefore, PANoptosis may constitute a novel therapeutic target in the treatment of IBD. 

#### PANoptosis and Pathophysiology of IBD

IBD is characterized by extensive inflammatory infiltration of the intestinal mucosa, an imbalance between pro- and anti-inflammatory cells, and alterations in intestinal barrier function. In recent years, studies in human tissue and animal models have shown that disruption of cell death pathways is a fundamental factor in the pathogenesis of the disease.

Regarding the molecular mechanisms underlying PANoptosis, it is argued that PANoptosis is mediated by the Panoptosome, also known as the Panoptisome. This complex includes the following key regulators of cell death: Z-DNA binding protein 1 (ZBP1), which represents a sensor of cellular stress and infections, RIPK1/RIPK3 corresponding to the central kinases of the necroptosis pathway, and Caspase-1/Caspase-8, which regulate pyroptosis and apoptosis. The ZBP1 is the master orchestrator of PANoptosis in the gut, via the IFN-ADAR1-ZBP1 Axis. Interferons (IFN) induce the expression of ZBP1. Under normal conditions, ADAR1 suppresses ZBP1 activation. However, in the inflamed gut, the loss of ADAR1 function or the presence of Z-RNA triggers ZBP1 to recruit RIPK3 and Caspase-8, leading to PANoptotic cell death [19]. Activation of the Panoptosome in IBD leads to the simultaneous release of inflammatory cytokines (via pyroptosis) and damage-associated molecular patterns (DAMPs) (via necrosis), creating a vicious cycle of inflammation [20]. The molecular mechanisms involved in the pathogenesis of IBD are associated with increased NLRP3 inflammasome activation. In IECs, PANoptosis leads to “shedding” and barrier breaches, facilitating bacterial translocation. In Lamina Propria Immune Cells, PANoptosis, and specifically the pyroptotic component, triggers the release of IL-1β and IL-18, thus amplifying the inflammatory cascade. 

**Figure 2:**
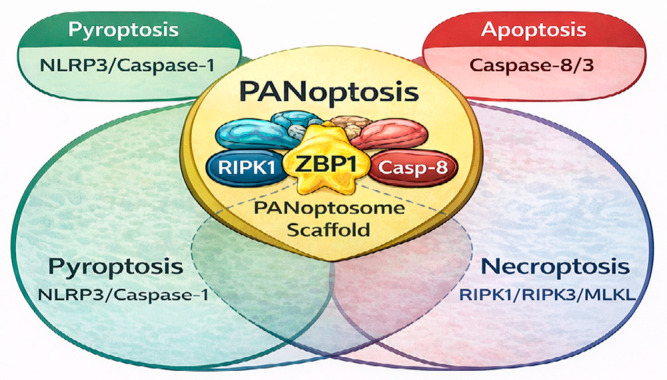
Crosstalk and convergence of programmed cell death pathways in PANoptosis. The Venn diagram illustrates the intersection of three distinct cell death modalities: Pyroptosis (NLRP3/Caspase-1), Apoptosis (Caspase-8/3), and Necroptosis (RIPK1/RIPK3/MLKL). PANoptosis represents the functional convergence of these pathways, primarily orchestrated by the PANoptosome scaffold and the sensor ZBP1. This integration explains why inhibiting a single pathway (e.g., necroptosis via RIPK1 inhibitors) may fail clinically, as the cell can switch to alternative PANoptotic routes.

Regarding differences between UC and CD, the two diseases present also differences in the mechanisms of cell death. Thus, in UC, intense induction of necrosis and inflammatory destruction of the mucosa is observed. In contrast, in CD, necroptosis activation is mainly observed in myeloid and epithelial cells, with greater involvement of RIPK3/MLKL. These differences also underpin the development of targeted drugs that regulate specific PANoptosis pathways for each disease. In more detail, in CD, it has been shown that PANoptosis of IECs promotes the inflammatory process. Zhao J et al. [20], in a recent study including in vitro and in vivo experiments, demonstrated that Intelectin-1 is overexpressed to a statistically significant degree in the IECs of inflamed intestinal mucosa in CD patients, and it is significantly correlated with inflammatory markers that increase in CD. In this way, they promote the inflammatory process and PANoptosis and weaken the tight junctions of IECs through binding to the protein calpain-2. The E3 ubiquitin ligase directly interacts with calpain-2, mediating its ubiquitination and degradation. At the same time, they showed that PANoptosis induced by the Z-DNA binding protein 1 antagonizes the PANoptosis-promoting protein calpain-2, demonstrating that Intelectin-1 improves colonic inflammation and intestinal barrier function in interleukin-10 knockout mice. Therefore, pharmacological inhibition of Intelectin-1 ameliorates epithelial cell damage and colitis both in vivo and in vitro, suggesting that therapies targeting Intelectin-1 and PANoptosis are promising strategies for the treatment of patients with CD [21]. Figure 2 shows the interplay of PANoptosis pathways in IBD.

Several genes are involved in the PANoptosis processes of IBD, e.g., OGT, TLR2, GZMB, TLR4, PPIF, YBX3, CASP5, BCL2L1, CASP6, MEFV, GSDMB, and BAX. These genes are specifically identified hub genes from transcriptomic models and do not represent the entire PANoptotic machinery. Zhang M et al. [21] showed that the previously mentioned genes are associated with TNF-α signaling, NF-κB, pyroptosis, and necroptosis, and that the nomogram model and calibration curves have substantial predictive value. They also found that immune cell infiltration was increased in patients with IBD, and the model genes were closely associated with infiltration by various immune cells. The transcription factors associated with DE-PRGs were RELA, NFKB1, HIF1A, TP53, and SP1. The findings of the study indicate that the DE-PRG model genes have satisfactory prognostic value in IBD, and that PANoptosis processes participate in the pathogenesis of the disease through TNF signaling, NF-κB, pyroptosis, necroptosis, and immune mechanisms [22]. Li Y et al. [22] analyzed transcriptomic data from patients with UC, CAC, and control groups to identify genes associated with PANoptosis. Four PANoptosis hub genes (CASP1, LCN2, STAT3, ZBP1) were identified as factors that favor the development of UC. Strong binding affinity between epidermal growth factor receptor inhibitors and target proteins was also found, suggesting therapeutic potential. The authors conclude that the PAR-Score based on PANoptosis genes predicts the progression of UC and the therapeutic response to anti-TNF-α therapy. EGFR inhibitors may serve as potential therapeutic agents for UC and the prevention of UC-associated colorectal cancer [23]. Yang Y et al. [23] showed that PANoptosis plays a vital role in CD by altering the immune system and interacting with CD-related genes. More specifically, in their study, they identified 10 PANoptosis-related genes with satisfactory diagnostic performance: CD44, CIDEC, NDRG1, NUMA1, PEA15, RAG1, S100A8, S100A9, TIMP1, and XBP1. These genes showed significant associations with certain immune cell types and CD-related genes [24]. Wan C et al. [24] in a recent study identified three PANoptosis-related genes, namely BIRC3, MAGED1, and PSME2, which were associated with neutrophils, CD8+ T cells, activated CD4 T cells, NK cells, IBD, as well as the IL-17 and NOD-like receptor signaling pathways [25]. Wang JM, et al. [25]. identified five key genes of PANoptosis (ZBP1, AIM2, CASP1/8, IRF1) associated with UC. They hypothesized that IRF1 promotes PANoptosome expression, activates PANoptosis, thus inducing IECs excessive death [26]. Ji Y, et al. [26] identified seven hub PANoptosis-related genes with potential diagnostic value for UC and suggested that PANoptosis contribute to the pathogenesis of UC by regulating specific immune cells and interacting with key signaling pathways [27]. Lu J, et al. [27] identified five key candidate genes, TIMP1, TIMP2, TIMP3, IL6, and CCL2, associated with PANoptosis in UC, with strong diagnostic performance [28].

Regarding CD, Chen Y et al. [28] recently tried to construct CD PANoptosis signature using Multiple Machine Learning Models, Molecular Subtyping and Singlecell Analysis. They found that CD PANoptosis signature consisted of seven genes: CEACAM6, CHP2, PIK3R1, CASP10, PSMB1, PSMB8 and UBC. Multiple analyses suggested that I-kappaB kinase/NF-kappaB signalling, mitogen-activated protein kinase, leukocyte activation and leukocyte migration were the mechanisms likely involved in PANoptosis in CD. Therefore, PANoptosis may mediate the process of CD through inflammatory and immune mechanisms, such as NF- kappaB, MAPK and leukocyte migration [29].

Regarding the role of Interferon-γ, it is known that it affects IECs in many ways, being overexpressed in the lamina propria of the intestinal wall. Lee C et al. [29], in a recent study using intestinal organoids (enteroids) derived from non-IBD controls and CD patients found that, IFN-γ induced PCD of enterocytes in both control and CD patient enteroids in a dose-dependent manner. All three processes, namely pyroptosis, apoptosis, and necroptosis, were activated in enteroids, suggesting that PANoptosis was the primary process of PCD induced by IFN-γ and that it was cell-type dependent on the epithelial enterocyte [30]. They also found that upadacitinib, a selective JAK1 inhibitor used in the treatment of IBD, effectively blocked IFN-γ-induced cytotoxicity and PANoptosis.

Multiple studies link the activation of markers across apoptosis, pyroptosis, and necroptosis to IBD, and combined signatures of these markers are increasingly reported in both experimental models and human IBD samples. However, the field is still defining how best to operationalize PANoptosis as a clinical biomarker or therapeutic target [27]. There is evidence linking PANoptosis-related gene expression and protein activation patterns to IBD pathophysiology, suggesting that this integrated form of cell death contributes to disease and might be detectable in patient tissues. However, research is still nascent compared to individual cell-death pathways, and much of the evidence comes from transcriptomics and integrated pathway analyses rather than large longitudinal clinical cohorts, A standardized PANoptosis biomarker panel for IBD diagnosis or prognosis has not yet been fully validated.

## 3. Pharmacological Targeting Strategies of PANoptosis 

The therapeutic potential of the PANoptosis target lies in its role as a unified PCD pathway that can be manipulated for IBD and other malignant and non-malignant disorders. Targeting PANoptosis involves developing drugs that can either induce it or eliminate inflammatory cells. Key targets include proteins within the PANoptosome complex, such as sensors like NLRP3 and ZBP1, and executioners like caspase-8, PIPK1/3, and gasdesmins (GSDM) [30,31]. 

This targeted regulation of PANoptosis constitutes a promising therapeutic approach, as it allows intervention across multiple molecular pathways involved in IBD pathogenesis. The main categories of pharmacological agents include necroptosis inhibitors, inflammasome inhibitors, anti-apoptotic regulators, immunobiological agents, and combination strategies [32,33]. Table 2 shows the pharmacological agents that target PANoptosis pathways.

Targeting PANoptosis requires inhibiting the central nodes that regulate the three types of death, as it is shown below [14].

### 3.1. RIPK1/2/3 and MLKL Inhibitors

RIPK1 and RIPK3 proteins are central regulators of necroptosis and participate in the formation of the necrosome. Pharmacological inhibition of RIPKs has emerged as a potential therapeutic route in IBD. Recent data highlight PP6 as a regulator of RIPK1-dependent PANoptosis, making it a possible new molecular intervention target [32]. Phosphoprotein analyses of patients with UC reveal MLKL/RIPK3 activation, suggesting a necroptosis pathway. Liu C et al. [37] recently described a mechanism in which P2Y14R modulates MLKL-dependent necroptosis and protects against colitis in experimental animal models [37]. Similar results were described by Lu et al. [38] showed in their experimental colitis study, they showed that RIPK1 inhibition protects tight junctions and reduces damage in DSS colitis [38]. Examples of such agents are:

- GSK2982772: This selective RIPK1 inhibitor was highly anticipated. However, recent Phase 2 clinical trial data showed that it did not achieve significant clinical response in patients with moderately to severely active UC. This highlights the redundancy of cell death pathways; when necroptosis is blocked, the cell may shift toward apoptosis or pyroptosis within the PANoptotic framework [34].
- Necrostatin-1 (Nec-1): one of the first RIPK1 inhibitors, with impressive activity in animal models (reduction in mucosal damage and cytokines).
- SZ-15, RIPA-56, and other newer small-molecule inhibitors.

Regarding the mechanism of action, RIPK1/3 inhibitors prevent the transcriptional activation of MLKL and, consequently, cell lysis through necroptosis, reducing the release of DAMPs and inflammatory factors. In more detail, Necrostatin-1 (Nec-1) is the most widely studied inhibitor of Receptor-interacting protein kinase 1 (RIPK1). RIPK1 is a central protein that functions as a “switch” between the survival/inflammation pathways (via NF-κB), apoptosis (via Caspase-8), and necrosis (via RIPK3/MLKL). The mechanism of action of Nec-1 is related to the fact that it acts as a stereoselective inhibitor of the RIPK1 kinase, preventing the formation of the necrosome (necrosome: RIPK1-RIPK3-MLKL). In this way, it (i) inhibits necrosis by achieving a reduction in inflammatory disruption of the cell membrane and (ii) reduces inflammation since RIPK1 participates in the activation of the NLRP3 inflammasome and therefore its inhibition indirectly reduces inflammation. The administration of Nec-1 or newer, more selective inhibitors of RIPK1 (Nec-1s or GSK’s inhibitors) in experimental colitis models in rats (DSS or TNBS colitis), the drug resulted in (i) a reduction in the Tissue Damage Index with a significant improvement in the clinical score (reduction in weight loss, bleeding), (ii) protection of the intestinal mucosa with an impressive reduction in IEC necrosis and improvement in the histological picture, (iii) a reduction in the levels of pro-inflammatory cytokines (TNF-α, IL-6 and IL-1β) in the intestinal tissue, and (iv) restoration of the mucosal barrier, contributing decisively to maintaining its integrity. It should be emphasized that, while Nec-1 primarily targets necrosis, RIPK1’s central role in regulating PANoptosis makes it a powerful tool for holistic intervention in cell death in IBD [14].

### 3.2. Inflammasome/NLRP3 Inhibitors

Inflammasome activation is a key process in PANoptosis, especially in UC. Targeting NLRP3 is considered one of the most promising therapeutic axes. Such pharmacological agents include MCC950 (NLRP3 inhibitor), which decreases IL-1β and IL-18 production, and CY-09 and β-caryophyllene, which have been shown to reduce IL-1β and IL-18 production in experimental colitis in mice [39,40,41]. While the role of the NLRP3 inflammasome is well-documented, clinical results have been sobering. Anakinra (IL-1R antagonist) and specific IL-18 inhibitors have failed to show significant clinical efficacy in general IBD populations, suggesting that blocking a single cytokine downstream of PANoptosis is insufficient.

### 3.3. Caspase Regulation

Pharmacological regulation of caspases can lead to either protection of the intestinal epithelium or inflammatory suppression of immune cells. Examples include Caspase-8 inhibitors, which, in experimental colitis models, reduce inflammation and IL-1β levels, and Pan-caspase inhibitors, which appear to limit epithelial damage.

### 3.4. IAPs/SMAC Mimetics

IAPs (Inhibitors of Apoptosis Proteins) negatively regulate apoptosis. SMAC mimetics are a newer class of drugs that reverse this action. Examples include the agents Birinapant and LCL161. Data in patients with IBD are mainly preclinical, but show the potential to reduce damage through the regulation of cell death and inflammation [42]. 

### 3.5. Anti-TNF and Other Biological Agents

Although anti-TNF agents were not designed to target PANoptosis, they do affect the pathway by modulating T-cell and macrophage apoptosis. Examples include Infliximab and Adalimumab, which induce lymphocyte apoptosis and reduce cytokine production. In addition, other biologic agents, such as ustekinumab and vedolizumab, modulate immune cell responses and may have a secondary effect on cell death. So far, there are some clinical/translational data suggesting that patients with IBD who do not respond to anti-TNF therapy may have increased pyroptosis associated with poor therapeutic outcomes. The available data indicate that increased pyroptotic signaling in the inflamed tissue is associated with non-response to anti-TNF therapy in IBD, and pyroptosis-related gene expression may have predictive biomarker potential, though these are mostly associative/translational rather than interventional clinical data. However, clear, large-scale clinical evidence linking necroptosis with anti-TNF non-response in patients is currently lacking or limited. The field is rapidly evolving, and biomarkers like pyroptosis gene signatures show promise for future clinical use [43]. 

### 3.6. Other Therapeutic Agents

Other therapeutic agents include dietary factors that affect the intestinal microbiota, agents that act on mitochondrial function, and plant products such as diasmin [14]. It has long been proven that IBD is pathogenetically associated with the presence of dysbiosis of the intestinal microflora. Dysbiosis involves the excessive growth of harmful microflora, accompanied by a parallel decrease in beneficial microflora, contributing, in synergy with other factors, to the onset of IBD. Various plant compounds have been shown to have beneficial effects on the course of IBD by improving or eliminating dysbiosis, reducing intestinal mucosal inflammation, and restoring increased intestinal barrier permeability. This is achieved through the regulation of signaling pathways such as TGF-β/Smad, TLR-4/NF-ΚB/MAPK, TLR2-NF-κB, autophagy, pyroptosis, glycolysis/gluconeogenesis, and amino acid metabolism; Nrf-2/HO-1; microbiota-macrophage-arginine metabolism; and bile acid metabolism, as well as through increased synthesis of short-chain fatty acids and other metabolites. However, the adverse effects of their excessive use, such as worsening of immune responses, should also be taken into account. Plant-based dietary compounds can contribute decisively to the treatment of IBD by modulating PANoptosis mechanisms [44]. Mitochondrial dysfunction results in the production of mitochondrial reactive oxygen species (mtROS), which negatively affect intestinal barrier function, by increasing intestinal mucosal permeability and promoting the inflammatory process through immune cell invasion. Therefore, targeting mtROS may be a therapeutic target. Gong W et al. [45] generated regulatory T cell (Treg)-derived exosomes loaded with selenium and the synthetic mitochondria-targeting SS-31 tetrapeptide, which binds mitochondria via a peptide linker that is cleaved by metalloproteinases. This actively targetable exosome-derived delivery system prevents mitochondrial reactive oxygen species production, thereby preventing PANoptosis. This exosome-delivery platform may be a promising therapeutic strategy for treating IBD [45]. Other natural products derived from plants, fruits, and vegetables have also been shown to regulate PCD [46]. Ye Z et al. [47] investigated the role of mitochondria in the development of UC using cellular and animal models, as well as clinical samples. Their results showed that IECs damage in experimental DSS colitis involves mitochondrial fission mediated by dynamin-related protein 1 (Drp1) and PANoptosis dependent on Z-DNA binding protein 1 (ZBP1). ZBP1-PANoptosis and Drp1-mediated mitochondrial fission were observed in patients with UC, depending on disease severity. Hyperactivated mitochondrial fission led to the production of mitochondrial oxygen radicals and the appearance of PANoptosis, independent of ZBP1’s Zα domain, through sulfenylation of Cys327. Saquinavir has also been shown to inhibit mitochondrial fission, thereby enhancing therapeutic efficacy in experimental colitis [47]. 

Diosmin is a naturally occurring flavonoid with anti-inflammatory and antioxidant properties. Diosmin have shown protective effects not just as general antioxidants, but by specifically modulating PANoptotic markers. Diosmin appears to inhibit mitochondrial oxidative stress and reduce the expression of ZBP1 and phosphorylated RIPK3 in DSS-induced colitis models. Yang X, et al. [48] recently described mechanism linking oxidative stress to intestinal inflammation [48]. Tan C et al. [49] investigated these properties of Diosmine in the DSS-induced colitis model and in an LPS-induced model in human colonic epithelial cells. They specifically focused on the effects of Diosmine on PANoptosis of IECs, intestinal microflora, and fecal metabolites. They found that Diosmine significantly improved colitis symptoms, and histopathological analysis confirmed reduced inflammation and tissue damage in mice treated with Diosmine. Diosmine also suppressed the expression of genes and proteins associated with PANoptosis (ZBP1 and Caspase-1), preserving the integrity of the epithelial barrier in vitro. Furthermore, it altered the composition of the intestinal microbiota, promoting beneficial taxa such as Ruminococcus and reducing pathogenic Proteobacteria [49]. These data suggest that Diosmine may be an essential therapeutic agent in patients with IBD, by inhibiting PANoptosis of IECs, preserving intestinal barrier function, and modifying the intestinal microbiota. Future therapies should focus on “PANoptosome” stabilizers or dual inhibitors that could prevent the “pathway switching” that often leads to treatment resistance.

### 3.7. Therapeutic Synergy: Combination Therapies

The main challenge in existing IBD treatments, particularly with biologic agents (mainly anti-TNF-α agents), is primary or secondary non-response or loss of response [50]. This failure is often associated with:

- Non-Caspase-dependent Death: TNF-α inhibitors function in part by promoting apoptosis (Caspase-dependent). If cell damage leads to Necrosis (RIPK1/MLKL), then anti-TNF therapy becomes ineffective.
- Uncontrolled Pyrolysis: Inflammation can be maintained by pyrolysis, which is not entirely blocked by targeting TNF-α and requires inhibition of the inflammasome (e.g., NLRP3/Caspase-1).

Combination therapies to target PANoptosis represent a field of increasing research. The strategy of combining a Panoptotic Inhibitor (e.g., Nec-1 or MCC950) with a Biological Agent (e.g., anti-TNF) provides a dual mechanistic advantage, as shown in Table 3.

The combination appears promising (i) in patients with refractory IBD, in whom the predominant pathology is cell necrosis, and (ii) in cases with high inflammatory burden, where simultaneous targeting of inflammation and systemic inflammation (TNF-α) may lead to faster and deeper remission. The proposed combinations with possible therapeutic synergies involve anti-TNF + RIPK1 inhibitor, SMAC mimetic + inflammasome blockers, and small-molecule modulators specifically designed for gut-restricted targeting (Table 4).

## 4. Discussion

Accumulating human data suggest that IBD is characterized by concurrent activation of multiple regulated cell-death pathways, consistent with the conceptual framework of PANoptosis. While most early work focused on apoptosis, more recent studies using human intestinal biopsies, patient-derived organoids, and transcriptomic datasets demonstrate simultaneous upregulation of pyroptotic, apoptotic, and necroptotic components within the same tissues.

Several transcriptomic analyses of colonic biopsies from patients with active IBD reveal co-enrichment of genes related to inflammasome activation (e.g., CASP1, GSDMD), apoptotic executioners (e.g., CASP3, CASP8), and necroptotic mediators (e.g., RIPK3, MLKL). Importantly, these signatures are observed in human samples, not only in murine models, supporting biological relevance to human disease. However, these studies typically infer PANoptosis indirectly, based on gene co-expression patterns rather than direct demonstration of a unified death complex.

At the protein level, immunohistochemical and immunoblot analyses of IBD biopsies show cleaved caspase-3, cleaved gasdermin-D, and phosphorylated MLKL within inflamed intestinal epithelium. The co-localization of these markers within the same tissue compartments suggests that epithelial and myeloid cells in IBD may undergo hybrid or overlapping death programs, rather than mutually exclusive pathways. This observation aligns with the PANoptosis paradigm, which posits coordinated engagement of pyroptosis, apoptosis, and necroptosis downstream of inflammatory cues.

Patient-derived intestinal organoids exposed to inflammatory cytokines (e.g., TNF-α and IFN-γ) further support this concept, as they display simultaneous activation of multiple death effectors, closely recapitulating findings in primary human tissue. Such systems strengthen the translational bridge between human disease and mechanistic interpretation.

Key regulatory nodes implicated in PANoptosis—such as ZBP1 and caspase-8—are increasingly reported to be upregulated in inflamed IBD mucosa. ZBP1 expression in particular correlates with epithelial stress and inflammatory signaling in human samples, suggesting that PANoptosome-like signaling platforms may be active in human intestinal inflammation. Nevertheless, direct biochemical evidence of a canonical PANoptosome in human IBD tissue is still lacking, and current conclusions remain inferential.

Despite growing support, several limitations temper the interpretation of PANoptosis in human IBD:

- Lack of a unique PANoptosis biomarker: PANoptosis is defined conceptually rather than by a single molecular marker, making definitive identification in patient samples challenging.
- Predominantly associative human data: Most human studies are cross-sectional and correlative, limiting causal inference between PANoptosis activation and disease initiation or progression.
- Heterogeneity of IBD: Inter-patient variability, disease location, treatment exposure, and inflammatory burden complicate uniform interpretation of PANoptosis signatures.
- Overlap with severe inflammation: It remains difficult to distinguish whether PANoptosis is a driver of pathology or a consequence of intense inflammatory stress in advanced disease.

In the near future, the most crucial development is the strategy of combination therapies. Monotherapy whether with biological agents that modulate cytokines or with small molecules that target a single cell death pathway are likely to lead to the emergence of escape mechanisms. The combination of a PANoptosis inhibitor (which blocks the source of inflammation) with a cytokine inhibitor (which controls systemic inflammation) represents a synergistic approach that may lead to deeper remission, higher rates of mucosal healing, and overcoming resistance to existing biologics. Future research must determine if ZBP1 can be safely targeted without compromising antiviral immunity, and whether PANoptosis inhibitors can work synergistically with anti-TNF agents to prevent epithelial “sloughing” and promote mucosal healing. Clinical trials should also focus on optimal dosing and on potential toxicity. The possible role of natural products in the prevention and treatment of IBD should not be overlooked, bearing in mind their minimal side effects.

## 5. Conclusions

In summary, current human evidence supports the involvement of PANoptosis-related signaling in IBD, as reflected by the concurrent activation of pyroptotic, apoptotic, and necroptotic markers in patient tissues. Transcriptomic, protein-level, and patient-derived ex vivo data collectively suggest that intestinal cells in IBD do not undergo isolated forms of cell death but instead engage integrated inflammatory death programs consistent with PANoptosis.

However, the evidence remains largely indirect and associative, and definitive proof of PANoptosis as a unified, regulated cell-death mechanism in human IBD is still incomplete. Future studies combining spatial multi-omics, single-cell proteomics, and functional assays in human tissue will be essential to validate PANoptosis as a bona fide pathogenic mechanism rather than a descriptive framework.

If confirmed, PANoptosis could provide a unifying explanation for epithelial barrier loss, immune activation, and treatment resistance in IBD, and may open the door to novel therapeutic strategies targeting shared upstream regulators rather than individual cell-death pathways. Therefore, targeting central regulators, such as RIPK1 (with agents such as Necrostatin-1), seem to protect the intestinal barrier in preclinical models. Furthermore, a combination strategy involving PANoptosis inhibitors (to address the cause of cell death) and established biologic agents (e.g., anti-TNF) (to control the cytokine storm) represents the most advanced therapeutic perspective. This approach is likely to lead to a new generation of therapies that will dramatically improve response rates and quality of life for patients with IBD.

## Figures and Tables

**Table 1:** Main Pathways of PANoptosis in IBD.

Pathway	Main Molecules	Role in IBD
Apoptosis	Caspase-8, Fas, TNF	Regulation of epithelial homeostasis, impaired in severe inflammation
Pyroptosis	NLRP3, Caspase-1, GSDMD	IL 1β/IL 18 secretion, worsening inflammation
Necroptosis	RIPK1, RIPK3, MLKL	Mucosal destruction, epithelial barrier disruption
PANoptosis	ZBP1, PANoptosome	Combined activation and enhancement of inflammation

**Table 2:** Drugs targeting PANoptosis pathways.

Agent	Target	Mechanism of Action	Status/ Outcome
GSK2982772	RIPK1	Necroptosis/ PANoptosis	Negative. Phase 2 trial failed to meet primary endpoints in UC. (*) [34]
Anakinra	IL-1R	Pyroptosis (Downstream)	Lack of Efficacy in standard IBD trials (**) [35,36].
Necrostatin-1	RIPK1	Necroptosis	Preclinical only; used as a tool compound.
ZBP1 Inhibitors	ZBP1	PANoptosis	Experimental. No clinical-grade inhibitors currently available for IBD.
Diosmin	ZBP1/Mito	Multi-pathway	Preclinical success; potential for adjunctive therapy.

(*): No significant differences in efficacy were observed between treatment groups [34]. (**): A clinical trial, presented only as an oral presentation (not as a full paper), revealed no benefit in patients with acute severe UC [35]. A case report in a complicated patient with severe UC showed good results [36].

**Table 3:** Suggested combinations of biological agent with PANoptosis inhibitors.

Agent	Main Function	Influence on IBD	Combined Advantage
Anti-TNF (Infliximab)	Cytokine inhibition (TNF-α)	Reduction in systemic and local inflammation.	It acts on inflammation.
Necrostatin-1 (Nec-1)	Inhibition RIPK1/Necrosis	Protection of the intestinal epithelium, interruption of the source of DAMPs.	It addresses the cause (cell death) and prevents resistance.
MCC950	Inhibition NLRP3/Pyroptosis	Reduction in IL-1β/IL-18, mucosal protection.	It inhibits the inflammatory response caused by cell death.

**Table 4:** Possible therapeutic synergies.

Combination	Target	Theoretic Benefit
Anti-TNF + RIPK1 inhibitor	Apoptosis + Necroptosis	Improving Response
JAK inhibitor + inflammasome inhibitor	Cytokines + Pyroptosis	Reducing resistance to treatment
IL-23 blocker + PANoptosis drug	Innate + acquired immunity	Reduction in mucosal Inflammation

## Data Availability

No new data were created or analyzed in this study.
